# Antibiotic use and irrational antibiotic prescriptions in 66 primary healthcare institutions in Beijing City, China, 2015–2018

**DOI:** 10.1186/s12913-021-06856-9

**Published:** 2021-08-18

**Authors:** Yumiti Taxifulati, Haishaerjiang Wushouer, Mengyuan Fu, Yue Zhou, Kexin Du, Xi Zhang, Yaoyao Yang, Bo Zheng, Xiaodong Guan, Luwen Shi

**Affiliations:** 1 Department of Pharmacy Administration and Clinical Pharmacy, School of Pharmaceutical Sciences, Peking University, Beijing, 100191 China; 2 International Research Center for Medicinal Administration (IRCMA), Peking University, No.38 Xueyuan Road, Haidian District, Beijing, 100191 China; 3 Institute of Clinical Pharmacology, Peking University First Hospital, Beijing, China

## Abstract

**Objectives:**

To identify the patterns of antibiotic use and irrational antibiotic prescriptions in primary healthcare institutions (PHIs) in Dongcheng District of Beijing, China.

**Materials and methods:**

All primary healthcare institutions (7 community healthcare centres and 59 community healthcare stations in total) in Dongcheng District were included in the study. Prescription data from January 2015 to December 2018 was derived from the *Beijing Prescription Reviewing System of Primary healthcare institutions* and analysed retrospectively. The antibiotic prescription rate was calculated and cases of irrational antibiotic prescriptions were identified.

**Results:**

We extracted 11,166,905 prescriptions from the database. Only 189,962 prescriptions were included in the study, among which 9167 (4.8%) contained antibiotics. The antibiotic prescription rate fell from 5.2% in 2015 to 4.1% in 2018 while irrational antibiotic prescription rate increased from 10.4 to 11.8%. Acute Bronchitis was the most prevalent diagnosis (17.6%) for antibiotic prescriptions, followed by Unspecified Acute Respiratory Tract Infection (14.4%), Acute Tonsillitis (9.9%), and Urinary Tract Infection (6.4%). Around 10% of the prescriptions for the top 7 diagnoses identified were rated as irrational. Cephalosporins, fluoroquinolones, and macrolides were the most prescribed antibiotics, which accounted for 89.3% of all antibiotic prescriptions. Of all the antibiotic prescriptions, 7531 were reviewed, among which 939 (12.5%) were rated as irrational because of antibiotic use. Among all the irrational prescriptions, prescriptions with inappropriate antibiotic use and dosage accounted for the majority (54.4%).

**Conclusion:**

Although a relatively low level of antibiotic utilization was found in PHIs in Dongcheng District of Beijing, the utilization patterns differed considerably from developed countries and irrational prescriptions remained. Considering the imbalanced allocation of medical resources between primary healthcare setting and secondary and tertiary hospitals, there need to be more efforts invested in regions with different levels of economic development.

**Supplementary Information:**

The online version contains supplementary material available at 10.1186/s12913-021-06856-9.

## Introduction

Antibiotic use is one of the major drives for antimicrobial resistance (AMR), a significant challenge to public health for all countries alike. China, a large consumer of antibiotics, has long suffered from irrational use of antibiotics and AMR. In the past two decades, the Chinese government has implemented a series of policies and measures to confront AMR, including restricting antibiotic use. The evolution of policy management has been well documented [1]. In 2018, China Antimicrobial Resistance Surveillance System revealed that the prevalence of commonly seen resistant bacteria continuously declined in the past 5 years, with methicillin-resistant *S. aureus* declined to 32.2%, carbapenem-resistant *P. aeruginosa* to 20.7%, and carbapenem-resistant *A. baumannii* to 56.1% [2]. Although there was no direct evidence drawing correlation between antibiotic use in the community with the prevalence of antibiotic resistance observed in hospitals, however, antibiotic use in primary health care was proved to affect AMR [3]. In recent years, studies have investigated antibiotic use in secondary and tertiary hospitals with various drug management programs to promote appropriate use of antibiotics [4–6]. These studies significantly increased after the implementation of the Zero Mark-up Policy, which remarkably reduced incentives for hospitals to rely on prescriptions for financial gains. Although the policies aimed to target all levels of healthcare institutions, however, most policies were implemented in secondary and tertiary hospitals, where more medical resources were allocated. Primary healthcare institutions thus did not proportionately benefit from the policies.

Moreover, information on antibiotic use and prescriptions in primary healthcare settings was missing due to issues of data accessibility. We identified one study that investigated patterns of antibiotic use in 48 primary healthcare facilities across China in 2014 [7]. Other that, we found little evidence about the patterns of antibiotic use and inappropriate antibiotic prescriptions in primary healthcare settings, which is important as primary healthcare institutions provided medical services to the majority of the population. According to the National Health Commission, out of 8.2 billion medical visits in China in 2017, 4.4 billion (54.2%) were in primary healthcare institutions [8]. Hence, a better understanding of antibiotic usage in primary healthcare institutions, especially the factors affecting inappropriate antibiotic use, is essential for key decision-making promote appropriate antibiotic use. To explore how antibiotics were used in primary healthcare institutions, we studied antibiotic prescriptions in primary healthcare institutions (PHIs) in Dongcheng District of Beijing between 2015 and 2018. We aimed to identify the frequency of antibiotics use and patterns of inappropriate antibiotic prescriptions.

## Methods

### Study setting and design

All PHIs in Dongcheng district of Beijing were included in the study, which consisted of seven community healthcare centres (CHCs) and 59 community healthcare stations (CHSs) during the study period. PHIs were the first point of contact of patients with the national healthcare system. All PHIs in Dongcheng District were outpatient-only clinics, responsible for providing basic outpatient clinical care and services to individuals and families residing in the community. We conducted a retrospective observational study to assess the appropriateness of antibiotic prescriptions in all PHIs covered by the Prescription Review Network in Dongcheng district, Beijing from January 2015 to December 2018.

### Data source

Prescription data was derived from the *Beijing Prescription Reviewing System of Primary healthcare institutions* (BPRSPHI), which was established by the Beijing Health Commission in 2014, collecting routine online prescription review of PHIs.

All outpatient prescriptions in BPRSPHI of the sample PHIs were collected. Data derived included prescribing date, patient age, gender, diagnoses, medications, and the prescription review result. Electronic prescriptions were digitally transferred from the database and double-checked by our researchers.

### Sampling process of BPRSPHI

A sampling software was embedded in PHIs’ information systems. No less than 1‰ of the total prescriptions were randomly selected by the sampling software and then reported to BPRSPHI from each PHIs monthly. One hundred prescriptions were randomly selected from each PHIs monthly, using a systematic sampling method, with sampling interval calculated as the number of total prescriptions divided by 100. The selection of antibiotic prescriptions was showed in Supplement 1.

### Reviewing process of BPRSPHI

According to “*Regulation Standard for Hospital Prescription Review*” issued by the Chinese Ministry of Health in 2010 [9], The prescription review process was conducted manually monthly by a reviewing team consisted of multidisciplinary healthcare professionals including physicians, pharmacists, microbiologists, as well as experts of medical management from tertiary hospitals. According to *Guideline for the prescription review process of Beijing healthcare institutions*, the review should be conducted based on the clinical pathways, medication, and clinical treatment guidelines, as well as the medication manufactory instructions. As quality control measures, the Hospital Medication and Therapeutic Committee was responsible for providing training for the reviewing team before the conduct of reviews, and the Medical Quality Committee was responsible for periodic quality evaluation after reviews.

### Patient and public involvement

No patients or members of the public were involved in this study.

### Definitions

A prescription in the study referred to all drugs prescribed for one patient during one visit. The antibiotics were classified according to Anatomical Therapeutic and Chemical (ATC) classification J01 (i.e., antibacterial for systemic use), as recommended by the WHO Collaborating Centre for Drug Statistic Methodology [10].

In this study, we only assessed and analysed the appropriateness of the antibiotic prescriptions. Antibiotic prescriptions were defined as the prescriptions that contained at least one antibiotic drug. According to *Regulation Standard for Hospital Prescription Review*, an irrational prescription is a prescription with writing and/or the clinical use of medication (including indication, selection of drugs, administration route, usage, and dosage, drug-drug interaction, and incompatibility of drugs) not conformed to relevant laws and technical specifications. An irrational prescription can be categorized as one of the following sub-types based on whether the use of antibiotics conformed to guidelines: irregular prescription, inappropriate prescription, and abnormal prescription. These sub-types differ by types of mistakes or inappropriateness of the prescription (detailed definition of the three types of irrational prescriptions were shown in Table 3) and were not mutually exclusive, as observed in some prescriptions categorized into multiple sub-types.

### Data analysis

The major indicators were selected based on WHO recommendations [11]. The antibiotic prescription rate was calculated by dividing the number of antibiotic prescriptions by the number of total sample prescriptions. The rate of irrational antibiotic prescription was calculated by dividing the number of irrational antibiotic prescriptions by the total number of antibiotic prescriptions. The dataset was managed in Microsoft Excel and computed in Stata (version 14.0). Continuous variables were expressed as mean and standard deviation (SD) and categorical data was expressed in proportions. The cost was converted from Chinese RMB into US dollars uniformly using an exchange rate of 6.89 yuan to 1 US dollar.

### Ethics and consent

Informed consent was waived by Peking University Institution Review Board. Ethics committee approval was obtained from Peking University Institution Review Board (IRB00001052–17016).

## Results

### Characteristics of the sample prescriptions

Of the total 66 community health care institutions included in the study, 189,962 prescriptions (1.7%) out of 11,166,905 prescriptions between January 2015 and December 2018 were extracted from the BPRSPHI. The characteristics of the sample prescriptions were described in Table 1. 28,217 (14.9%) and 161,745 (85.1%) of the total prescriptions were from CHCs and CHSs respectively. Of the 28,217 prescriptions issued in CHCs, 9167 (4.8%) contained antibiotics, among which 7531 prescriptions had a review result and 12.5% were rated as irrational due to inappropriate antibiotic use. Almost all antibiotics prescribed were non-injective and antibiotics accounted for 1.9% of the total cost of prescribed drugs. The proportion of prescriptions containing antibiotics demonstrated a downward trend from 2.4% in 2015 to 1.6% in 2018, while the irrational antibiotic prescription rate remained higher than 10% during the study period.

**Table 1:** Characteristics of prescriptions from all the primary healthcare institutions in Dongcheng District covered by Beijing Prescription Reviewing System of Primary healthcare institutions, 2015–2018

Characteristics	2015			2016			2017			2018			Total		
	CHCs	CHSs	All	CHCs	CHSs	All	CHCs	CHSs	All	CHCs	CHSs	All	CHCs	CHSs	All^**a**^
**No. of PHIs**	7	48	55	7	55	62	7	52	59	7	57	64	7	59	66
**Total prescriptions, n**	467,108	1,447,744	1,914,852	593,995	1,454,909	2,048,904	749,082	1,724,979	2,861,411	906,122	1,955,289	2,474,061	7,944,603	3,222,302	11,166,905
**Sample prescriptions from BPRSPHI, n**	8455	11,580	20,035	7120	52,943	60,063	6835	50,120	56,955	5807	47,102	52,909	28,217	161,745	189,962
**Drugs prescribed per prescription, mean (SD)**	1.8 (1.1)	1.9 (1.1)	1.8 (1.1)	2.1 (1.2)	1.9 (1.1)	1.9 (1.1)	2.1 (1.2)	2.0 (1.1)	2.0 (1.1)	2.0 (1.2)	2.0 (1.2)	2.0 (1.2)	2.0 (1.2)	1.9 (1.1)	1.9 (1.1)
**Cost per prescription, $ mean (SD)**	19.2 (27.8)	18.4 (25.5)	18.8 (26.5)	25.2 (33.5)	20.0 (28.6)	20.6 (29.2)	27.3 (37.1)	24.4 (34.7)	24.8 (35.0)	28.3 (38.1)	27.6 (37.8)	27.6 (37.8)	24.6 (34.0)	23.5 (33.4)	23.6 (33.5)
**Antibiotic-containing prescriptions, % of prescriptions**	5.8	4.8	5.2	7.5	4.9	5.2	5.5	4.9	5.0	6.1	3.9	4.1	6.2	4.6	4.8
**Prescriptions with antibiotic injections, % of antibiotic prescriptions**	0.2	0.0	0.1	0.6	0.0	0.1	0.0	0.0	0.0	0.0	0.1	0.0	0.2	0.0	0.1
**Cost of antibiotics in the total cost of prescribed drugs, %**	2.7	3.0	2.4	2.5	3.6	2.3	2.0	2.7	1.8	1.7	2.8	1.6	2.1	3.0	1.9
**Irrational antibiotic prescriptions, % of antibiotic-containing prescriptions**^**a**^	10.3	10.9	10.4	11.6	15.5	14.8	12.8	10.6	10.9	11.9	11.8	11.8	11.5	12.7	12.5

Abbreviations: *CHCs* community healthcare centres, *CHSs* community healthcare stations, *BPRSPHI* Beijing Prescription Reviewing System of Primary healthcare institutions

^a ^The denominator was 7531 antibiotic prescriptions after excluding those without expert opinions

### Antibiotic use in PHIs

Table 2 illustrated the characteristics of antibiotic prescriptions. Of all 9167 antibiotic prescriptions, 1754 (19.1%) and 7413 (80.9%) prescriptions were from CHCs and CHSs respectively. Patients aged 18–60 (43.1%) were the population most prescribed with antibiotics, followed by patients aged 61–70 (29.4%). More female patients (60.2%) were prescribed antibiotics. 1.3% of the antibiotic prescriptions contained more than one antibiotic. Antibiotic prescriptions during winter seasons (29.3%) were slightly more frequent than other seasons. Acute Bronchitis was the most prevalent diagnosis (17.6%) for antibiotic prescriptions, followed by Unspecified Acute Respiratory Tract Infection (14.4%), Acute Tonsillitis (9.9%), and Urinary Tract Infection (6.4%). When looking at the sub-type of irrational prescriptions of 7531 reviewed antibiotic prescriptions, most irrational prescriptions (87.1%) were rated as inappropriate (inappropriate antibiotic usage and dosage” accounted for 54.4% of all inappropriate antibiotic prescriptions). The frequency of the three sub-types of irrational prescription of sample antibiotic prescriptions was demonstrated in Table 3.

**Table 2:** Characteristics of antibiotic prescriptions among all the sample prescriptions in primary healthcare institutions in Beijing, 2015–2018

Characteristics	CHCs (%)	CHSs (%)	All (%)
**Total**	**1754 (100.0)**	**7413 (100.0)**	**9167 (100.0)**
**Year**			
2015	490 (27.9)	551 (7.4)	1041 (11.4)
2016	535 (30.5)	2576 (34.8)	3111 (33.9)
2017	376 (21.4)	2450 (33.1)	2826 (30.8)
2018	353 (20.1)	1836 (24.8)	2189 (23.9)
**Age**			
18–60	818 (46.6)	3137 (42.3)	3955 (43.1)
61–70	488 (27.8)	2209 (29.8)	2697 (29.4)
71–80	277 (15.8)	1216 (16.4)	1493 (16.3)
81–90	159 (9.1)	761 (10.3)	920 (10)
> 90	12 (0.7)	90 (1.2)	102 (1.1)
**Gender**			
Female	1085 (61.9)	4429 (59.8)	5514 (60.2)
Male	669 (38.1)	2984 (40.3)	3653 (39.9)
**Number of prescribed antibiotics**			
1	1724 (98.3)	7326 (98.8)	9050 (98.7)
≥ 2	30 (1.7)	87 (1.2)	117 (1.3)
**Season**			
Spring	405 (23.1)	1611 (21.7)	2016 (22)
Summer	470 (26.8)	1684 (22.7)	2154 (23.5)
Autumn	412 (23.5)	1903 (25.7)	2315 (25.3)
Winter	467 (26.6)	2215 (29.9)	2682 (29.3)
**Conditions**			
Acute bronchitis	314 (17.9)	1296 (17.5)	1610 (17.6)
AURI, not specified	246 (14)	1072 (14.5)	1318 (14.4)
Acute tonsillitis	40 (2.3)	866 (11.7)	906 (9.9)
Urinary tract infection	145 (8.3)	439 (5.9)	584 (6.4)
Paradentitis	120 (6.8)	458 (6.2)	578 (6.3)
Common cold	64 (3.6)	452 (6.1)	516 (5.6)
Acute pharyngitis	148 (8.4)	309 (4.2)	457 (5)
Other infection	107 (6.1)	307 (4.1)	414 (4.5)
Combined infection	570 (32.5)	2214 (29.9)	2784 (30.4)
**Irrational use**^**a**^			
Irregular	51 (26.7)	100 (13.4)	151 (16.1)
Inappropriate	155 (81.2)	663 (88.6)	818 (87.1)
Abnormal	2 (1)	14 (1.9)	16 (1.7)

Abbreviations: *CHCs* community healthcare centres, *CHSs* community healthcare stations, *AURI* Acute Respiratory Tract Infection

^a^ The denominator was irrational antibiotic prescriptions

**Table 3:** Frequency of three types of irrational prescription of sample antibiotic prescriptions, 2015–2018

No.	Irrational prescription type	Frequency ***N*** = 1049	Proportion %
**1**	**Irregular prescription**	***n*** **= 153**	**100.0**
1–1	The physician didn’t prescribe antibiotics follow the *Regulations on the Clinical Application of Antibiotics.*	98	55.1
1–2	Prescribing without clinical diagnosis or with incomplete clinical diagnosis.	33	18.5
1–3	Elements were missing in the prescriptions, non-standard or illegible writing	14	7.9
1–4	The dosage, specifications, quantity, unit, etc. of the drugs were not standardized or unclear.	3	1.7
1–5	The expression of dosage or usage was ambiguous, such as “follow the doctor’s advice”, “self-medicated”, etc.	2	1.1
1–6	The physician didn’t follow the related guidelines when prescribing narcotic, psychotropic, medical toxic, radioactive drugs, etc.	2	1.1
1–7	No reason stated for dosage over 7 days for outpatient, over 3 days for emergency, and extension for chronic diseases and geriatric disease	1	0.6
**2**	**Inappropriate prescription**	***n*** **= 880**	**100.0**
2–1	Inappropriate usage and dosage	571	64.9
2–2	Inappropriate indication	144	16.4
2–3	Inappropriate dosage form or route of administration	16	1.8
2–4	Inappropriate selection of drugs	63	7.2
2–5	Inappropriate combined use of drugs	39	4.4
2–6	Incompatibility or adverse interaction	25	2.8
2–7	Other inappropriate situation	16	1.8
2–8	Repeated administration	5	0.6
2–9	National essential medicines were not preferred without appropriate reason.	1	0.1
**3**	**Abnormal prescription**	***n*** **= 16**	**100.0**
3–1	Prescribing without indication	15	93.8
3–2	Prescribing high-priced drugs without appropriate reason.	1	6.3
3–3	Off-label drug use without appropriate reason.	0	0.0

As shown in Table 4, second-generation cephalosporins, fluoroquinolones, and macrolides were the most prescribed antibiotics in both CHCs and CHSs, accounting for 89.3% of all antibiotic prescriptions. 6.1% of the antibiotics were combinations of a β-lactamase inhibitor plus penicillin, mostly amoxicillin-clavulanate. Among the most prescribed antibiotics in 2018, only two out of ten were ranked in the same sequence as in 2015. The antibiotic prescribing pattern was similar between CHCs and CHSs.

**Table 4:** Top 10 prescribed antibiotic classes in primary healthcare institutions in Beijing, 2018

ATC Code	Antibiotic class	Antibiotic Rx in 2018, n (%)			Ranking in 2015
		CHC ***n*** = 455	CHS ***n*** = 2159	All ***n*** = 2614	
J01DC	Second-generation cephalosporins	144 (38.2)	1029 (52.0)	1173 (49.8)	1
J01MA	Fluoroquinolones	104 (27.6)	316 (16.0)	420 (17.8)	3
J01FA	Macrolides	56 (14.9)	296 (15.0)	352 (14.9)	2
J01CR	Penicillin plus β -lactamaseinhibitors	19 (5.0)	124 (6.3)	143 (6.1)	4
J01DD	Third-generation cephalosporins	30 (8.0)	70 (3.5)	100 (4.2)	10
J01XD	Imidazoles	14 (3.7)	71 (3.6)	85 (3.6)	5
J01DB	First-generation cephalosporins	0 (0.0)	62 (3.1)	62 (2.6)	6
J01XX01	Fosfomycin	6 (1.6)	8 (0.4)	14 (0.6)	9
J01JB	Aminoglycosides	2 (0.5)	3 (0.2)	5 (0.2)	7
J01FF	Lincosamides	2 (0.5)	0 (0.0)	2 (0.1)	7

Abbreviations: *CHCs* community healthcare centres, *CHSs* community healthcare stations

**Fig. 1:**
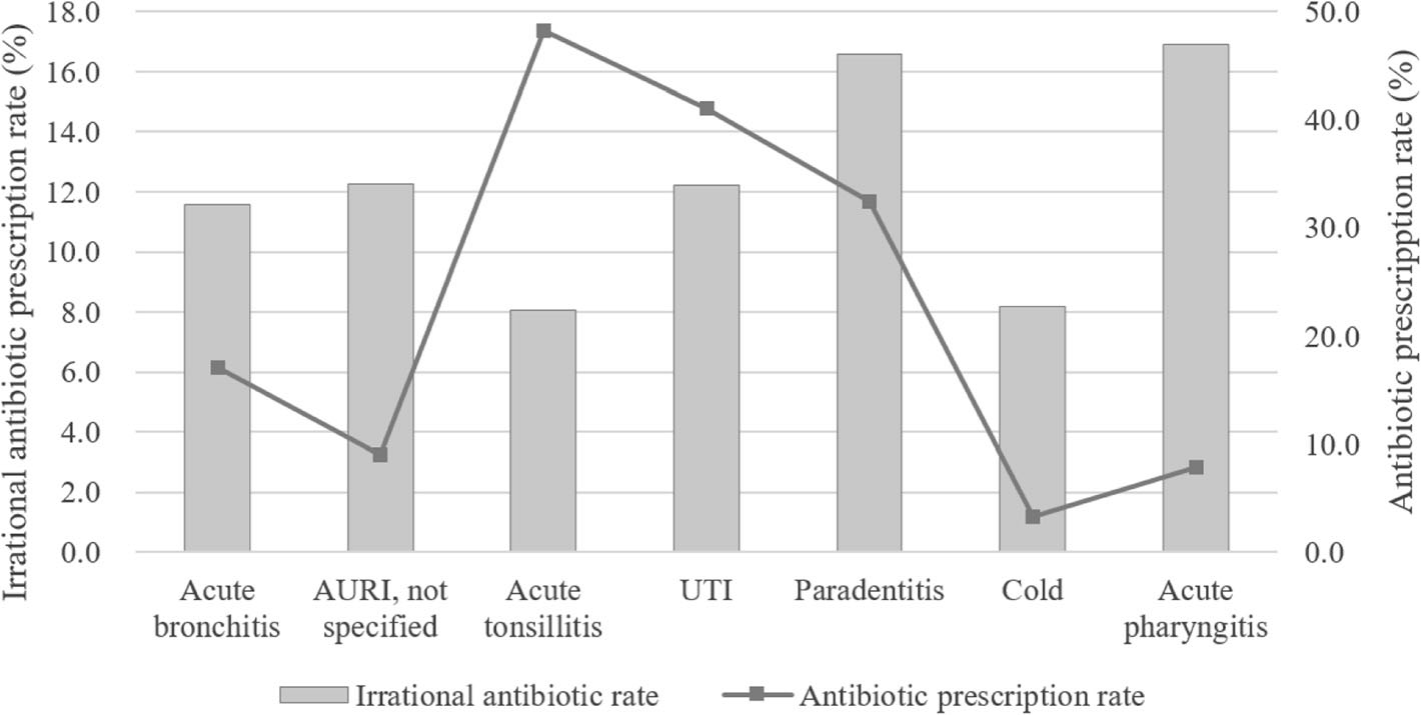
Antibiotic prescription and irrational prescription rate and in the most common conditions among all the sample prescriptions in PHIs in Beijing, 2015–2018. * Abbreviations: AURI, Acute Respiratory Tract Infection; UTI, Urinary Tract Infection

Antibiotics prescriptions, as well as irrational antibiotic prescriptions for the most common conditions, were presented in Fig. 1. Antibiotics prescription rate ranged from 3.3% (Common Cold) to 48.3% (Acute Tonsillitis) for these seven conditions. The most prevalent conditions for irrational antibiotic prescribing were Acute Pharyngitis (16.9%) and Paradentitis (16.6%).

## Discussion

Our study identified the patterns in antibiotic use and irrational antibiotic prescriptions in primary health care settings in Dongcheng District, Beijing, using prescription data from a total of 66 Primary Healthcare Institutions. According to the National Health Commission, the outpatient antibiotic prescription rate in Chinese hospitals (secondary and tertiary hospitals) declined from 16.2% in 2011 to 7.7% in 2017. Similarly, the inpatient antibiotic prescription rate also declined from 55.2 to 38.0% [2]. As for the primary healthcare setting in China, a previous study indicated that between 2009 and 2011, the outpatient antibiotic prescription rate was 52.9% in China and only 39.4% of the antibiotic prescriptions were considered appropriate [7]. Although the types of antibiotics that were prescribed in the primary healthcare setting were similar to those in secondary and tertiary hospitals, excessive and improper use of antibiotics was more common in primary care settings: the outpatient antibiotic prescription rate in PHIs ranged from 24.1 to 50.0% in Hubei, Shandong, and Jiangsu, with irrational prescription rate ranged from 30.1 to 40.8% [12–15]. In our study, the antibiotic prescription rate in PHIs was 6.7%, with very few antibiotic injections prescribed. In terms of prescribing appropriateness, the overall irrational antibiotic prescription rate in our study was relatively low (8.7%) compared with other parts of the country [12–15]. As Beijing is the capital city of China, this relatively low rate of antibiotic prescription rate in community settings could be explained by enhanced healthcare management that the city enjoys. The authority initiated a wide implementation of prescription review in PHIs since 2014, as well as establishing assisting mechanisms by which physicians were periodically sent from higher-level hospitals to PHIs to help improve care delivery of healthcare providers in PHIs. However, since Dongcheng District was one of the core districts of Beijing, with abundant medical resources, patients could seek healthcare services by skipping PHIs and directly visiting higher-level medical institutions, which could be a potential confounding factor that affects the result of the study.

Internationally, the antibiotic prescription rate in primary care settings was relatively high in developing countries and particularly in rural areas, with the rate being over 50% in Malaysia, India, and South Korea [16–18]. This rate was much lower in developed countries. The antibiotic prescription rate for Acute Respiratory Tract Infections was 14.7% in the United States and 33.0% in the UK [19, 20]. In our study, despite that the antibiotic prescription rates for Acute Pharyngitis and Common Cold were low (9.7 and 8.5% respectively), antibiotics were still prescribed for 20.9% of the patients with Acute Bronchitis, which exceeded the 20% recommendation made by the European Surveillance of Antimicrobial Consumption for patients with Acute Upper Respiratory Infection [21]. To address this issue, a new guideline for THE primary care of acute tracheobronchitis has been released by the Chinese Medical Association, recommending no antibiotic treatment for patients with uncomplicated Acute Tracheobronchitis without pneumonia. However, primary care providers might have difficulties accessing updated clinical guidelines as most guidelines were published in academic journals and cannot be obtained for free, resulting in a poor guideline compliance rate [22]. For elderly patients, antibiotic treatment was to be considered only when the patient aged over 80 and had one of the following conditions: orally given glucocorticoid, diagnosed with diabetes or congestive heart failure; or aged over 65 and had two of the conditions heretofore mentioned [19]. In addition, our study showed that cephalosporins were the most prescribed antibiotics in PHIs. This preference was also observed in general hospitals in China [2] and echoed antibiotic use in communities settings of European countries, where penicillins and tetracyclines were most commonly prescribed [21]. One of the possible reasons was that skin testing was an mandatory requirement for penicillin before administration (regardless of route) in China [23], which might underlie physicians’ preference of quinolones and cephalosporins to accommodate for time constraints.

The most commonly seen sub-type of irrational prescriptions was inappropriate prescriptions, among which “inappropriate antibiotic usage and dosage” was the most prevalent issue, accounting for more than 60% of all inappropriate prescriptions (Table 3). When looking at these irrational antibiotic prescriptions, we found some common patterns. First, over-dosage was the most frequently seen irrational prescription, for instance, cefuroxime axetil was prescribed more than 0.25-g t.i.d instead of b.i.d a day for patients with Acute Bronchitis. Second, incompatibility was neglected when it came to patients with multiple conditions. For instance, there were many circumstances where azithromycin was prescribed together with statins for patients with dyslipidaemia, which would increase the risk of myopathy. Other commonly seen irrational antibiotic prescriptions were the ones with unspecified indication towards antibiotics. Studies showed that the lack of expertise of physicians about antibiotics was one of the most important reasons for the irrational use of antibiotics in many countries [24, 25]. As medical resources were disproportionately distributed in China, where secondary and tertiary hospitals were allocated with more resources than PHIs, how to facilitate the flow of medical resources towards primary care facilities is one of the major tasks of the government. This requires a collaborative effort targeting from medical education to career development of the physicians, engaging also qualified pharmacists in treating patients to ensure appropriate antibiotic use. Compared with developed countries, pharmacists were less involved in clinical practices in China [26]. Especially in PHIs, the pharmacists as the gatekeeper for patients’ medication was not well enforced [27]. Last but not the least, it is strongly recommended that efforts should be made to incorporate primary healthcare institutions into the national surveillance network of antibiotic use and AMR. This could help the government comprehend the antibiotic usage and the prevalence of AMR more accurately, facilitating the decision-making in tackling AMR [28].

Our study had several limitations. First, Dongcheng is a unique district in the centre of Beijing with better economic development and medical resources. Thus, the study sample institutions were not fully representative of all PHIs in Beijing. Second, the socio-economic background of the patients was not analysed due to issues in data accessibility, which might introduce bias to the analysis. However, since the elder population accounted for the majority of patients in primary healthcare settings in our study, the impact of patient flow might not be determinant. Third, because the prescription reviewing process was conducted based on prescription per visit instead of each patient, the linkage between prescriptions with patients was not assessible. Nonetheless, the analysis based on prescriptions was accurate enough to reflect the patterns in antibiotic usage in PHIs. Forth, the sample prescriptions in BPRSPHI only accounted for a small proportion of the total prescriptions in all PHIs. Although the sampling process was based on a systematic randomized methodology, we could not avoid the potential selection bias during the reporting process. However, the random selection process could maximize the representativeness of the prescriptions. Fifth, the quality control of the data in BPRSPHI might have varied since its establishment in 2014, with improvements to the database were made every year. Nevertheless, we dropped the data in 2014 to minimize the impact of the discrepancy in data quality.

## Conclusion

Although a relatively low level of antibiotic utilization was found in the primary healthcare settings in Dongcheng District of Beijing, the utilization patterns differed considerably from developed countries, with a proportion of irrational prescriptions remained. Targeting inappropriate antibiotic use in primary healthcare settings is crucial to attaining the overall goal of rational antibiotic use in China. Considering the disproportionate allocation of medical resources between primary healthcare setting and secondary and tertiary hospitals, there need to be more efforts invested in regions with different levels of economic development.

## Supplementary Information

**Additional file 1.**

## Data Availability

The data that support the findings of this study are available from the Health Commission of Dongcheng, Beijing but restrictions apply to the availability of these data, which were used under license for the current study, and so are not publicly available. Data are however available from the authors upon reasonable request and with permission of the Health Commission of Dongcheng, Beijing.
