# Supplementary figures and images for: Antibiotic use and irrational antibiotic prescriptions in 66 primary healthcare institutions in Beijing City, China, 2015–2018
Source: BMC Health Serv Res. 2021 Aug 18;21:832. doi: 10.1186/s12913-021-06856-9 (PMC8371863; doi:10.1186/s12913-021-06856-9)

Legend: **The selection of antibiotic prescriptions**


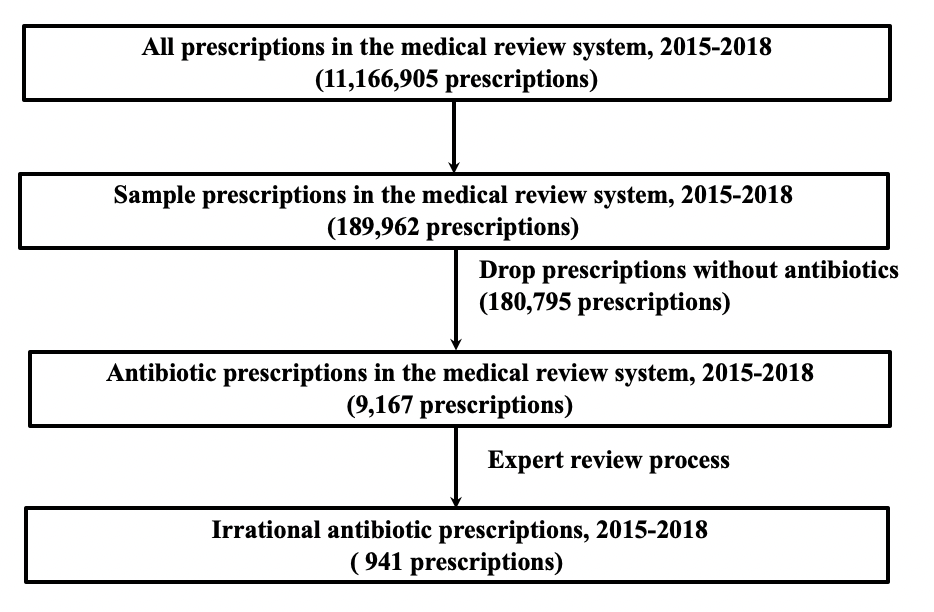

Supplement: Supplementary file 1 — Additional file 1. [file 12913_2021_6856_MOESM1_ESM.docx]
